# Nitrogen Regulation Under Heavy Metal Stress in Soil–Plant Systems: Implications for Plant Growth and Phytoremediation

**DOI:** 10.3390/plants15193065

**Published:** 2026-10-08

**Authors:** Ruize Sun, Asma Farooq, Sijin Chen, Aitezaz Ali Asad Shahani, Ting Liu, Li Jiajin, Taimoor Hassan Farooq, Wende Yan, Jun Wang

**Affiliations:** 1 Changsha Technology Innovation Center for Territorial Spatial Ecological Restoration, Central South University of Forestry and Technology, Changsha 410004, China; 19563358969@163.com (R.S.); asmafarok000@gmail.com (A.F.); lt19900501@163.com (T.L.); csfuywd@hotmail.com (W.Y.); 2 Bangor College, Central South University of Forestry and Technology, Changsha 410004, China; sjc23ghg@bangor.ac.uk (S.C.); jjl23bbh@bangor.ac.uk (L.J.); 3 College of Agriculture, Yangzhou University, Yangzhou 225009, China; 2193609022@ybu.edu.cn

**Keywords:** heavy metal pollution, nitrogen metabolism, phytoremediation, soil amendments, nutrient management, sustainable agriculture

## Abstract

Nitrogen (N) is a vital macronutrient that dictates soil fertility and plant productivity. Heavy metal contamination threatens plant health by disrupting nutrient uptake and photosynthesis. Specifically, heavy metals accumulate in soils and inhibit key N-assimilatory enzymes, such as nitrate reductase. This inhibition restricts N availability, which limits protein synthesis and reduces overall plant biomass. This review synthesizes the recent literature to evaluate the mechanisms of heavy metal–N interactions, highlighting a critical scientific insight: N exerts a dual role in heavy metal stress. Externally, the chemical form of N uptake alters rhizosphere pH, which can either mobilize or precipitate toxic metal cations. Internally, optimal N assimilation fuels the synthesis of metal-chelating proteins (e.g., phytochelatins) required for cellular detoxification. A key conclusion of this review is that isolated agronomic or physiological interventions are insufficient to manage this stress in the field. Effective mitigation requires a coupled “soil-to-gene” approach. By combining macroscopic soil management (e.g., pH buffering and biochar application) to restrict external metal bioavailability with targeted molecular interventions (e.g., regulating root ion transporters and leveraging microbiome-assisted multi-omics) to enhance internal detoxification, researchers can improve phytoremediation efficiency and restore soil health in multi-contaminated environments.

## 1. Introduction

Nitrogen is a key macronutrient required for chlorophyll synthesis, protein production, and overall biomass accumulation [1,2]. Plants primarily absorb N in the form of nitrate (NO_3_^−^) and ammonium (NH_4_^+^), which are assimilated into organic molecules essential for growth. However, heavy metal contamination severely impairs N uptake and assimilation, exacerbating heavy metals’ toxicity and limiting plant growth. The dual role of N in both enhancing and mitigating heavy metal toxicity adds complexity to these interactions, making it essential to understand the underlying mechanisms. The interaction between heavy metals and N is influenced by factors such as soil pH, heavy metal concentration, and plant species [3]. Heavy metals can bind to N assimilatory enzymes, disrupting their functionality and leading to metabolic imbalances. Additionally, heavy metals can alter soil N transformation processes, such as mineralization and nitrification, further limiting N availability for plants. These interactions have important implications for plant health, agricultural productivity, and ecosystem stability.

To understand these interactions, it is necessary to define heavy metals. Heavy metals are naturally occurring elements with a specific density greater than 5 g/cm^3^ [4]. In plant physiology, these elements are categorized by their biological function. Plants require trace amounts of specific heavy metals, such as zinc (Zn), copper (Cu), and molybdenum (Mo), to sustain basic metabolic processes. Molybdenum is specifically required for the plant N cycle. It forms the molybdenum cofactor (Moco), which binds to the apoenzyme of nitrate reductase (NR). Nitrate reductase requires Moco to catalyze the reduction of nitrate to nitrite during primary N assimilation [5,6]. Without adequate Mo, nitrate accumulates in plant tissues and N assimilation stops.

However, non-essential heavy metals, including cadmium (Cd), mercury (Hg), and lead (Pb), have no biological function. They cause toxicity even at low concentrations [7,8,9]. Human activities, such as mining, smelting, and the application of agrochemicals, increase the concentration of these non-essential metals in soils [10]. When plants absorb these toxic metals, they compete with essential elements for enzyme binding sites and generate reactive oxygen species (ROS). This disrupts cellular functions and decreases crop yields [11].

A critical aspect of heavy metal toxicity is its impact on N metabolism. Heavy metals interfere with N assimilation by directly disrupting enzymatic activities. For example, cadmium (Cd) inhibits nitrate reductase (NR) activity through two primary biochemical mechanisms. First, Cd has a high binding affinity for the sulfhydryl (-SH) groups of cysteine residues located in the enzyme. Binding to these groups alters the enzyme’s three-dimensional conformation and blocks the internal electron transfer from NADH to the active site [12]. Second, Cd disrupts the molybdenum cofactor (Moco) domain, either by displacing the essential Mo atom or by degrading the cofactor structure [13,14]. This dual inhibition stops the reduction of nitrate to nitrite. Consequently, nitrate accumulates in plant tissues, and the plant experiences severe N starvation despite adequate soil N levels. These biochemical disruptions create a cycle of nutrient limitation and oxidative stress that decreases agricultural productivity.

To mitigate the adverse effects of heavy metals–N interactions, several strategies have been proposed: Soil amendments: The application of lime, biochar, and organic matter to reduce heavy metals bioavailability and improve soil health. Phytoremediation: The use of hyperaccumulator plants to extract and stabilize heavy metals in contaminated soils. Nutrient management: Optimizing N fertilization to enhance plant growth and reduce heavy metals uptake. Microbial interventions: Utilizing N-fixing bacteria and other microbes to enhance N availability and heavy metals detoxification. These strategies aim to alleviate heavy metals toxicity, improve plant productivity, and restore soil fertility, contributing to sustainable agricultural practices and ecosystem resilience.

While previous reviews address heavy metal toxicity and plant metabolism, they often treat these as separate systems. For example, Hossain et al. [15] reviewed heavy metal-induced oxidative stress and glutathione-mediated detoxification, and Zhao et al. [16] summarized general heavy metal uptake and crop improvement strategies for food safety. These reviews do not address how soil N management directly alters internal N assimilation under heavy metal stress. This review fills that gap. We synthesize the recent literature to show how external soil N dynamics, such as pH changes driven by nitrate versus ammonium uptake, couple with internal molecular responses, including nitrate reductase inhibition and amino acid synthesis. By linking soil chemistry to plant genetics, this review provides a specific framework for using N regulation to improve phytoremediation and crop growth in contaminated soils.

This review highlights the mechanisms of heavy metals–N interactions, their physiological and biochemical consequences, and potential mitigation strategies, providing a comprehensive framework for addressing this critical issue. Addressing the challenges posed by heavy metals pollution and its interaction with N requires interdisciplinary collaboration among researchers, policymakers, and stakeholders. By integrating knowledge from plant science, soil science, and environmental engineering, we can develop innovative solutions to mitigate heavy metals toxicity, enhance N use efficiency, and promote sustainable land management practices.

## 2. Literature Selection

To identify studies examining the interactions between heavy metals and N in plants, we conducted a literature search across three databases: Web of Science (Core Collection), Scopus, and PubMed. The search included articles published between January 2000 and August 2026. We used Boolean operators to combine three main concept groups: heavy metal stress, N metabolism, and mitigation strategies. The exact search string applied across all databases was: (“heavy metal” OR “cadmium” OR “lead” OR “mercury”) AND (“Nitrogen assimilation” OR “ Nitrogen metabolism” OR “nitrate reductase” OR “ Nitrogen fixation”) AND (“phytoremediation” OR “soil amendment” OR “plant breeding” OR “genetic engineering”).

This search strategy identified an initial pool of 210 records after removing duplicates. We then screened (175) these records based on their titles and abstracts to determine their relevance to the core themes of this review. We assessed study quality based on methodological criteria. Articles were included in the final review if they met three specific conditions: (1) they used controlled experimental designs (e.g., hydroponic or soil-grown plants with defined HM exposure concentrations and durations); (2) they measured specific N-related physiological or molecular variables (e.g., nitrate reductase activity, N uptake rates, or gene expression); and (3) they reported appropriate statistical analyses to support their claims.

**Figure 1:**
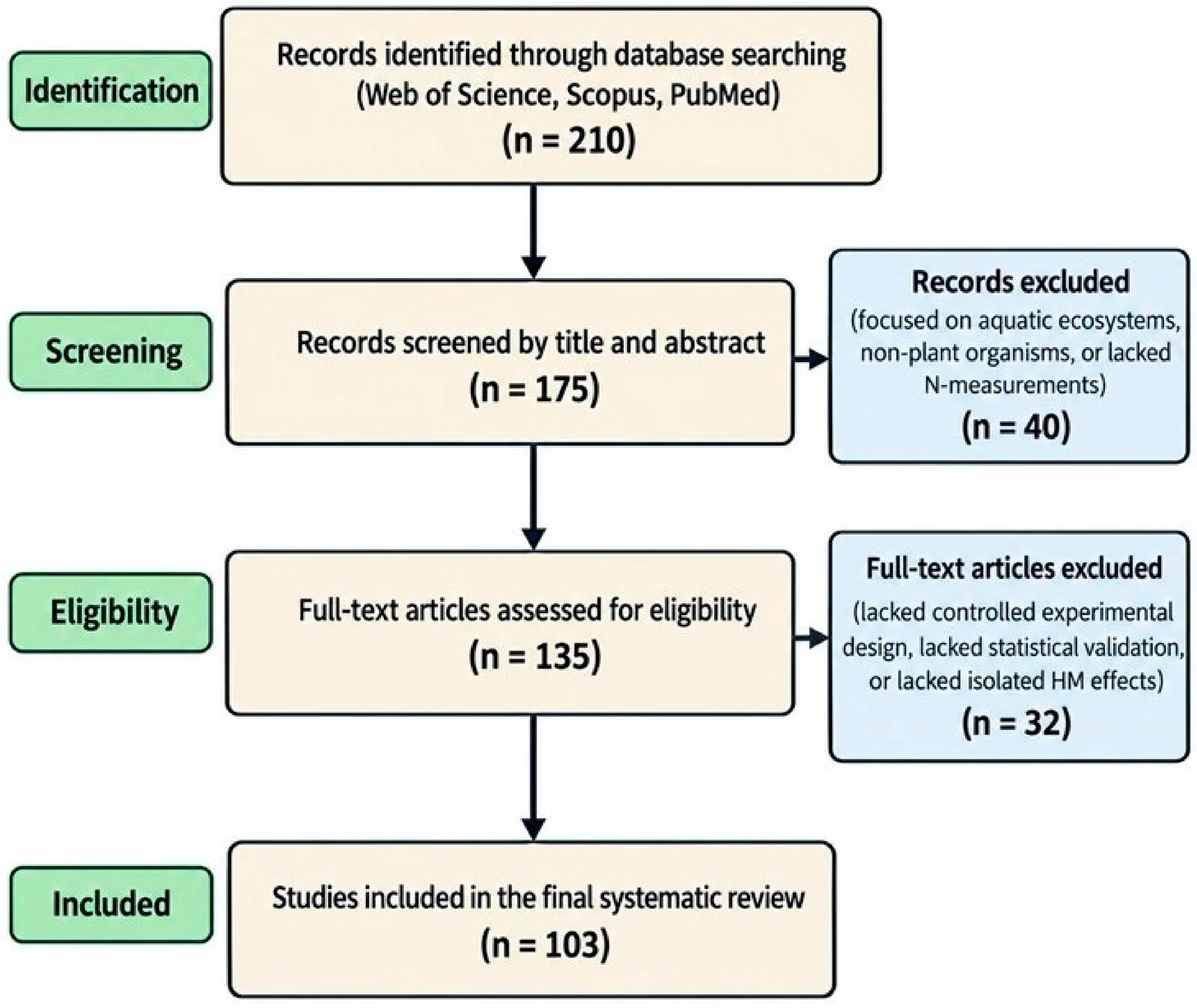
PRISMA-style flow diagram showing the identification, screening, eligibility assessment, and inclusion of studies in the systematic review.

Studies were excluded if they lacked adequate experimental controls, did not isolate the effects of heavy metals from other concurrent abiotic stresses, or lacked clear statistical methods. Applying these inclusion and exclusion criteria reduced the pool of 135 full-text articles to a final selection of 103 studies. We also incorporated relevant technical reports, review papers and grey literature specifically when discussing the practical, field-scale applications of soil amendments and phytoremediation (Figure 1).

## 3. Sources of Heavy Metals in the Environment and Impacts

**Figure 2:**
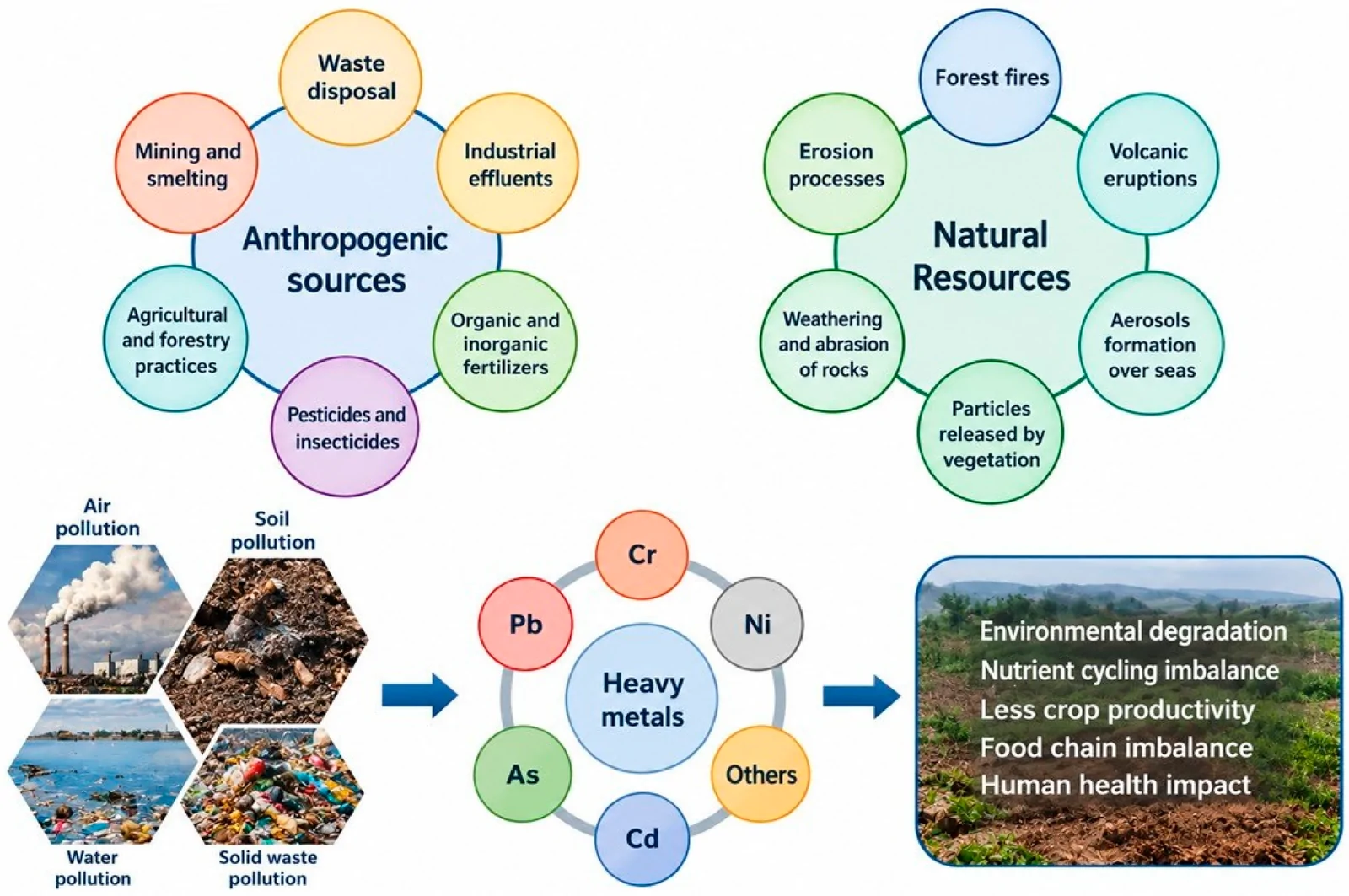
Sources of heavy metal pollution, categorized into anthropogenic (human-made) and natural origins. This figure illustrates the variety and significance of these sources in contributing to heavy metal contamination in the environment.

Heavy metals are persistent environmental pollutants known for their ability to accumulate and resist degradation in ecosystems. They are commonly found in forms such as hydroxides, phosphates, oxides, sulfides, silicates, carbonates, and organic complexes. These metals originate from both natural and anthropogenic sources [10] (Figure 2). While natural processes like weathering, volcanic eruptions, decay, and seismic activity contribute to their presence, human activities such as mining, smelting, industrial operations, and metal-based manufacturing are the primary drivers of heavy metals contamination in the environment [10,17,18].

The toxicity of heavy metals is often linked to their density and chemical properties, with even metalloids like arsenic posing significant risks at low exposure levels. In recent years, the widespread use of heavy metals in industrial, agricultural, domestic, and technological applications has led to a dramatic increase in environmental contamination and human exposure. Sources of metalloids and HMs include geogenic processes, industrial discharges, agricultural runoff, pharmaceutical waste, domestic effluents, and atmospheric deposition [17].

While this review primarily focuses on true heavy metals, it also includes the metalloid arsenic (As) due to its frequent co-occurrence in contaminated agricultural soils. However, arsenic has different chemical properties, environmental speciation, and uptake mechanisms compared to metallic elements like Cd, Pb, Cu, and Zn. In soil solutions, true heavy metals typically exist as positively charged cations (e.g., Cd^2+^, Pb^2+^). Because of this positive charge, plant roots absorb them through specific metal cation transporters, such as the ZIP and NRAMP families. In contrast, arsenic primarily exists as oxyanions: arsenate (AsV) in aerobic soils and arsenite (AsIII) in anaerobic soils, such as flooded rice paddies. Because arsenate is a chemical analog of phosphate, plant roots absorb it through high-affinity phosphate transporters. Arsenite, being a neutral molecule at typical soil pH, enters roots primarily through aquaporin channels (nodulin 26-like intrinsic proteins) [16]. Once inside the plant, their toxicological behaviors also diverge. While cationic heavy metals often displace essential cofactors in enzymes, arsenate specifically competes with phosphate in ATP synthesis, disrupting cellular energy metabolism.

Heavy metals affect the physiology and biochemistry of both plants and animals. While they play essential roles in oxidation-reduction reactions, their excess causes toxicity. For example, copper (Cu) is an essential cofactor in Cu/Zn-superoxide dismutases (Cu/Zn-SOD) and cytochrome c oxidase. In contrast, catalases and plant class III peroxidases are heme-containing enzymes that rely on iron (Fe). Beyond these redox enzymes, Cu connects directly to N assimilation. Cu is required for the function of the CNX1G protein domain, which catalyzes the insertion of molybdenum into the molybdenum cofactor (Moco) [5]. Because nitrate reductase requires Moco to function, Cu availability directly influences the plant’s ability to assimilate N. However, when Cu accumulates beyond homeostatic limits, the redox cycling between Cu(II) and Cu(I) generates hydroxyl radicals. This overaccumulation causes cellular and tissue damage, leading to adverse health effects in humans and plants alike [19].

## 4. The Role of Nitrogen in Heavy Metal Uptake, Toxicity, and Homeostasis

The influence of N on the uptake of heavy metals by plants is multifaceted and depends on several factors, including plant species, the type of heavy metals, soil N availability, and soil pH [20,21]. In many cases, N enhances the uptake of certain heavy metals by driving the synthesis of specific metal-chelating molecules and transporters. N assimilation provides the amino acid precursors (such as methionine) required to synthesize phytosiderophores (e.g., mugineic acid) and nicotianamine. Plant roots exude these molecules into the rhizosphere, where they bind and solubilize heavy metal cations [22].

Furthermore, this uptake is controlled by specific signaling pathways. Nitrate (NO_3_^−^) acts as a signaling molecule; its assimilation generates nitric oxide (NO), which upregulates the expression of Yellow Stripe-Like (YSL) transporter proteins on the root plasma membrane. These YSL transporters then actively absorb the soluble metal-phytosiderophore complexes from the soil into the root cells [23]. Additionally, N can improve heavy metals absorption by altering cell membrane permeability, facilitating the entry of metals into plant cells [24]. However, N can also reduce the uptake of certain heavy metals under specific conditions. For instance, N can elevate soil pH, rendering some heavy metals less soluble and thus less available for plant uptake [25]. Furthermore, N can suppress the activity of chelating agents, which typically bind to heavy metals and enhance their availability to plants [26,27,28].

### 4.1. Mechanisms of Heavy Metal Uptake in Plants: Facilitated Transport and Chelation

Plants absorb heavy metals from their environment through several mechanisms, including facilitated uptake, active transport, and diffusion. Among these, facilitated uptake is the most prevalent, relying on metal ion transporters to bind and transport metal ions into plant cells [29,30]. This process is crucial for the uptake of essential metals like iron (Fe) and Zn, which are vital for plant growth and development [31,32]. Facilitated uptake is supported by the following mechanisms: Metal Ion Transporters: These membrane-bound proteins bind to HM ions and facilitate their entry into plant cells. They can also function as ion pumps, actively transporting metals across cell membranes [33].

Once inside the cell, heavy metals are managed by distinct cytosolic proteins and enzymes. Metallothioneins (MTs) are cysteine-rich proteins located in the cytosol that directly bind metal cations. Phytochelatin synthase (PCS) is a cytosolic enzyme that synthesizes phytochelatins (PCs) from glutathione. These PCs then bind free heavy metals to neutralize their immediate toxicity. Glutathione-S-transferases (GSTs) are enzymes that facilitate the conjugation of glutathione to various toxic compounds, reducing oxidative damage [15]. None of these components act as ion pumps. Instead, after MTs and PCs bind the heavy metals in the cytosol, actual membrane-bound transport proteins, such as Heavy Metal ATPases (HMAs) and ABC transporters, pump the metal-chelate complexes across the tonoplast and into the vacuole for safe storage [34]. Uptake of heavy metals and plant responses to this heavy metals in different crops is shown in Table 1.

### 4.2. Nitrogen’s Influence on Heavy Metal Toxicity in Plants

Nitrogen plays a critical role in regulating the uptake and toxicity of heavy metals in plants. As an essential nutrient for growth and development, N influences a plant’s ability to absorb, store, and transport heavy metals, thereby modulating their toxicity [3,42]. Under severe N-deficient conditions, plants generally exhibit reduced overall metabolic activity and lower transpiration rates. This physiological downregulation consequently reduces the transpiration-driven mass flow of heavy metals into the roots, lowering the immediate risk of acute metal toxicity, albeit at the cost of stunted plant growth [43,44,45].

Conversely, high N application rates can inadvertently enhance heavy metal uptake and accumulation. Excessive nitrogen stimulates rapid vegetative biomass production and increases stomatal conductance. This increased water movement draws a higher volume of dissolved heavy metals from the soil solution into the root vascular system [46,47]. While high N levels initially promote faster growth, the subsequent hyperaccumulation of metals ultimately exacerbates toxicity symptoms, such as leaf senescence, root necrosis, and chlorosis, compared to plants grown in low-N soils [48]. These findings highlight the dual role of N in both mitigating and exacerbating heavy metal toxicity, depending on its availability and the plant’s transpiration dynamics.

### 4.3. N’s Role in Heavy Metal Homeostasis

By binding to heavy metals, N can reduce their bioavailability and toxicity [49]. Additionally, N stimulates the production of detoxifying enzymes, such as glutathione-S-transferases (GSTs), which bind to heavy metals and neutralize their harmful effects [15]. Furthermore, N enhances the synthesis of proteins like metallothioneins (MTs), which sequester HMs and prevent cellular damage [50]. One way in which N aids in heavy metal homeostasis is by upregulating specific metal transport and detoxification genes.

For example, N upregulates genes involved in metal transport and detoxification, one-way N aids in heavy metal homeostasis is by increasing plant tolerance to these metals through targeted gene expression. For example, N upregulates the iron efflux transporter gene OsFPN1 (Ferroportin 1) in rice, which maintains cellular iron homeostasis and prevents heavy metal-induced iron deficiency [51]. Similarly, in wheat, N application increases the expression of specific manganese transport genes, such as TaMTP8. This gene encodes a Metal Tolerance Protein that sequesters excess manganese into the vacuole, preventing cytosolic toxicity [16]. Table 2 summarizes the specific genes involved in N-mediated heavy metal transport and detoxification.

Moreover, N improves the plant’s capacity to absorb and utilize heavy metals by promoting the production of metal-chelating transporters. These proteins facilitate the transport of metal ions into plant cells, where they can be used in metabolic processes [52]. For instance, N has been shown to enhance the expression of metal uptake and utilization genes in wheat, improving its ability to manage heavy metals stress [53]. By boosting the production of detoxifying enzymes and metal-binding proteins, N helps plants maintain heavy metals homeostasis and mitigate toxicity.

### 4.4. Nitrogen Uptake and Transport in Plants and Heavy Metal Interference

**Figure 3:**
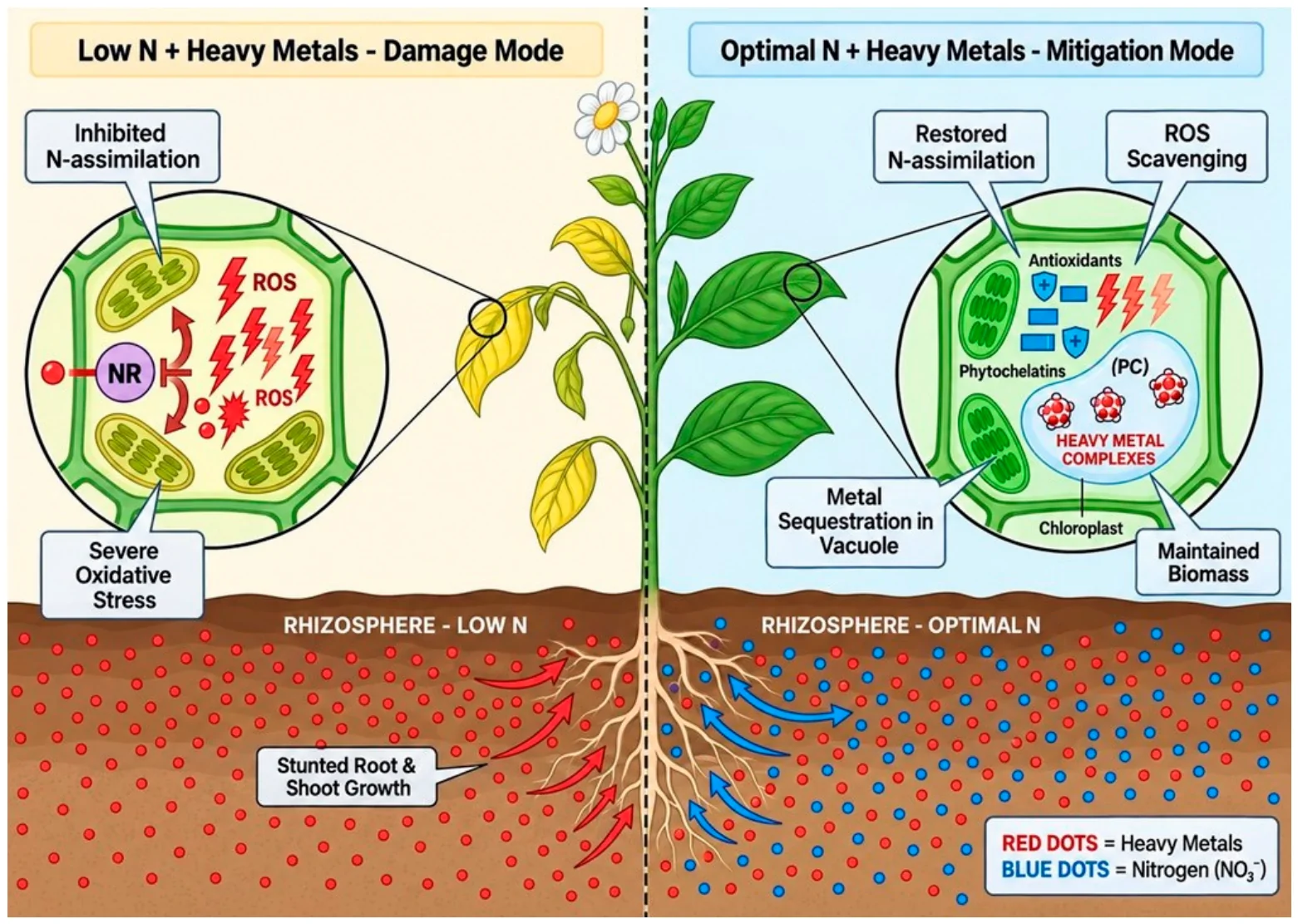
Comparative physiological and cellular responses to heavy metal stress under low versus optimal N regimes. (**Left**) Low N + Heavy Metals: Under N deficiency, heavy metals (red dots) accumulate rapidly in root and shoot tissues. At the cellular level, heavy metals inhibit nitrate reductase (NR) activity and trigger severe oxidative stress via the overproduction of reactive oxygen species (ROS). This results in chloroplast degradation, visible leaf chlorosis, and stunted biomass. (**Right**) Optimal N + Heavy Metals: Adequate N supply (blue dots) restores N-assimilation and fuels the plant’s internal detoxification machinery. Nitrogen drives the synthesis of antioxidants, which scavenge ROS, and phytochelatins (PCs), which chelate free heavy metal cations. These metal-PC complexes are subsequently sequestered into the vacuole, protecting cellular organelles and maintaining overall plant growth.

Nitrate (NO_3_^−^) is the primary source of N in aerobic agricultural soils, with its levels typically exceeding those of ammonium (NH_4_^+^). Even at high soil nitrate concentrations, plants actively absorb nitrate, as demonstrated by thermodynamic analyses [54]. Physiological studies have revealed that the influx of nitrate into root cells is closely linked to the activity of plasmalemma H^+^-ATPase, which pumps protons (H^+^) into the cells, creating a proton gradient that drives nitrate uptake [55]. Nitrate transport systems in plants are diverse: some are constitutively expressed, while others are induced by nitrate availability and regulated by its assimilation products (Figure 3).

At the molecular level, nitrate uptake relies on two main protein families: NRT2 and the broader NPF (NRT1/PTR) family. Historically, researchers classified NRT1 proteins exclusively as low-affinity transport systems (LATS) and NRT2 proteins as high-affinity transport systems (HATS) [56]. However, this classification oversimplifies their biological roles. NRT1 proteins belong to the NPF family, which exhibits diverse transport properties and regulatory functions. For example, specific NPF members, such as NRT1.1, function as dual-affinity transporters. They switch between low- and high-affinity states depending on external nitrate concentrations and their phosphorylation status [57]. Furthermore, NPF proteins transport other molecules, including peptides and plant hormones, which integrates N uptake with overall stress signaling. These systems enable plants to adapt to varying nitrate concentrations in the soil. However, the presence of heavy metals in the soil can severely disrupt nitrate uptake. Excessive heavy metal concentrations inhibit the activity of these nitrate transporters, impairing the plant’s ability to absorb and utilize nitrate [27].

To understand the molecular link between heavy metals like Mo and N assimilation, researchers rely on the green alga *Chlamydomonas reinhardtii*. *C. reinhardtii* is the reference organism for eukaryotic molybdenum metabolism. Studies on this alga led to the discovery of the first plant protein capable of binding the molybdenum cofactor (Moco) [6]. This Moco-binding protein protects the cofactor from oxidation and facilitates its insertion into the apoenzyme of nitrate reductase.

Furthermore, research in *C. reinhardtii* shows that molybdenum and nitrate homeostasis are genetically and physiologically linked. This relationship occurs through two novel ABC family transporters [58]. These transporters regulate the intracellular accumulation of molybdate in response to nitrate availability. When nitrate levels change, the expression of these ABC transporters adjusts, ensuring the cell has enough Mo to synthesize Moco for nitrate reductase. This mechanism indicates that plants and algae actively coordinate heavy metal uptake with macronutrient availability to maintain metabolic balance.

## 5. Interaction of Heavy Metals with Nitrogen Fixing Process

Biological N fixation accounts for approximately 70% of the elemental N used by plants. This process relies entirely on the Nase enzyme complex. Nase requires the iron-molybdenum (FeMo) cofactor to reduce atmospheric N to ammonia [59]. Therefore, biological N fixation depends directly on Mo availability and homeostasis in the nodule. Heavy metal stress decreases N fixation through both direct and indirect mechanisms. Directly, toxic heavy metals like Cd and Pb inhibit the survival and proliferation of free-living and symbiotic diazotrophs (e.g., Rhizobium) in the soil. Furthermore, toxic metals can directly interfere with Nase assembly by displacing essential metals like Fe or Mo in the FeMo-cofactor, rendering the enzyme inactive.

Indirectly, heavy metals reduce N fixation by damaging the host plant. Because N fixation requires high amounts of energy, any heavy metal-induced reduction in host photosynthesis decreases the carbon supply delivered to the root nodules. Additionally, heavy metal accumulation in the host plant increases ROS production within the nodules [60]. High ROS levels oxidize leghemoglobin, which normally protects the oxygen-sensitive Nase enzyme. Without functional leghemoglobin, Nase degrades, and N fixation stops.

### 5.1. Effect of Heavy Metals on Nitrogen Fixing Process

Heavy metals inhibit N fixation at the molecular level by targeting the Nase enzyme complex and its regulatory genes. The structural components of Nase are encoded by the nif gene cluster, with the nifH gene serving as a primary biomarker for N-fixing potential. Exposure to toxic metals, such as Cd and Pb, directly downregulates the transcription of nifH, reducing the synthesis of the Nase enzyme. Furthermore, heavy metals interfere with the post-translational assembly of the enzyme. Nase requires the iron-molybdenum (Fe-Mo) cofactor to function. Because Cd and Pb share similar ionic properties with essential transition metals, they compete with Fe and Mo during cofactor assembly. This displacement results in a misfolded, catalytically inactive Nase complex, halting the reduction of atmospheric N to ammonia.

The ecological impact of this inhibition extends beyond agricultural legumes. In non-agricultural and forest soils, N inputs rely heavily on free-living diazotrophs (e.g., Clostridium, Beijerinckia, and diverse uncultured bacterial taxa). Heavy metal contamination from mining runoff and atmospheric deposition severely alters these unmanaged ecosystems. For example, Zhao et al. [61] reported that high concentrations of soil Cd and Pb significantly reduced the abundance and diversity of the nifH gene in forest soils surrounding mining areas. This reduction indicates that heavy metals eliminate sensitive free-living diazotrophs, shifting the soil microbiome toward metal-tolerant but non-N-fixing species. Consequently, heavy metal pollution limits natural N inputs, which restricts plant regeneration and slows secondary succession in degraded forest ecosystems.

Even among tolerant microbial strains, heavy metal toxicity alters N-fixing efficiency. While some free-living bacteria, such as specific strains of *Azotobacter chroococcum*, exhibit tolerance to moderate heavy metal stress, their Nase activity still decreases under high Cd exposure due to the metabolic energy diverted toward metal efflux pumps rather than N fixation [62].

### 5.2. Symbiotic and Non-Symbiotic Relationship

Both symbiotic and non-symbiotic systems have undergone significant changes in N fixation due to HM stress. According to Hayat et al. [63], Cd stress resulted in a 34% reduction in root N content and symbiotic processes compared to the control group. The study noted a decrease in the quantity of nodules per plant and the dry mass of nodules, correlating with lower N fixation and reduced N levels in the roots. Furthermore, Cd toxicity has the effect of diminishing nodule N fixation by elevating the formation of ROS in nodules. This heightened ROS production leads to oxidative damage in the nodules [60,61,62,63,64].

Another heavy metal, As, has been found to significantly decrease N fixation in legumes and the activities of various N-fixing microorganisms. These reductions have been associated with root hair damage, leading to a shorter infected root zone. In the case of black gram, exposure to arsenic toxicity resulted in a twofold decrease in Nase activity within the nodules, consequently constraining the efficiency of N fixation. Despite the *Bradyrhizobium japonicum* E109 strain demonstrating tolerance to arsenic in soybean, nodulation was still adversely affected. This reduction was correlated with the decreased motility of the microorganism in the presence of arsenic [63].

## 6. Mitigation of Heavy Metal–N Interaction

### 6.1. Phytoremediation Prospects

**Figure 4:**
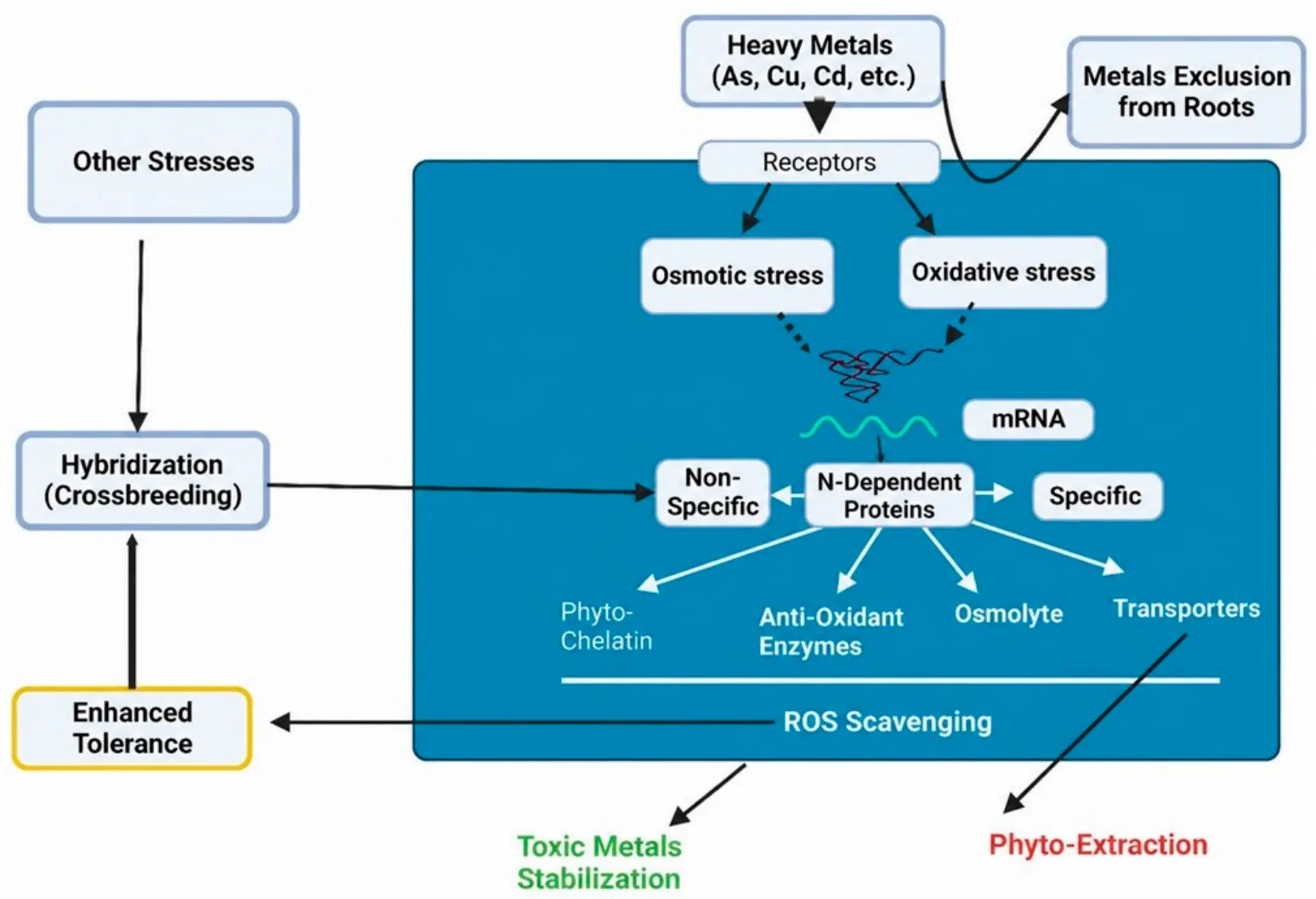
Genetic and molecular pathways driving heavy metal tolerance and phytoremediation. Heavy metal exposure triggers membrane receptors, initiating signaling cascades that respond to osmotic and oxidative stress. The subsequent transcription of mRNA translates into specific N-dependent proteins, including phytochelatins, antioxidant enzymes, osmolytes, and metal transporters. These proteins scavenge reactive oxygen species (ROS) and facilitate the stabilization or phyto-extraction of toxic metals. As shown on the left, traditional hybridization (crossbreeding) strategies leverage these endogenous pathways, combining traits from different stress responses to generate crop varieties with enhanced, multi-stress tolerance.

Phytoremediation uses plants to mitigate heavy metal contamination in soils. This approach encompasses two fundamentally different objectives: phytoextraction and phytostabilization [65] (Figure 4). Phytoextraction removes metals from the soil. In this process, plants absorb heavy metals through their roots and translocate them into harvestable above-ground tissues. Researchers then harvest and remove these plant tissues from the site, effectively extracting the pollutants [66].

In contrast, phytostabilization immobilizes metals within the soil matrix. To reclaim polluted soil, plants extend their root systems and alter rhizosphere chemistry. This process reduces metal mobility and bioavailability, preventing the metals from leaching into groundwater or entering the food web, without actually removing the metals from the site [67,68]. Both strategies allow plants to control heavy metal toxicity, but they achieve site remediation through different biological mechanisms.

Phytoremediation offers several practical advantages. First, it is economically feasible because it relies on solar energy, which keeps installation and maintenance costs low. Second, it reduces the environmental spread of pollutants. Third, plants stabilize heavy metals in the root zone, which prevents soil erosion and stops metals from leaching into groundwater [25,68,69].

Table 3 summarizes these macroscopic interventions across various major crops. The purpose of this table is to outline practical mitigation method, such as the application of organic fertilizers, targeted soil amendments, and crop rotation, that reduce heavy metal bioavailability and restore N utilization without requiring genetic modification. These agronomic approaches provide immediate, deployable solutions that complement the longer-term molecular breeding strategies discussed later in this review.

In addition to higher plants and N-fixing bacteria, soil microalgae contribute to heavy metal remediation. For example, the green microalga *Chlamydomonas reinhardtii* absorbs heavy metals from the soil solution. Once inside the cell, the alga sequesters these metals using intracellular chelating mechanisms to prevent cellular damage [79]. Furthermore, soil microalgae actively assimilate inorganic N from the soil. This assimilation temporarily stores N in algal biomass, which reduces N leaching in degraded soils. When the algae die and decompose, they release this N back into the rhizosphere, improving nutrient availability for plant roots. Therefore, integrating microalgae into phytoremediation strategies increases metal stabilization and supports soil N cycling.

**Figure 5:**
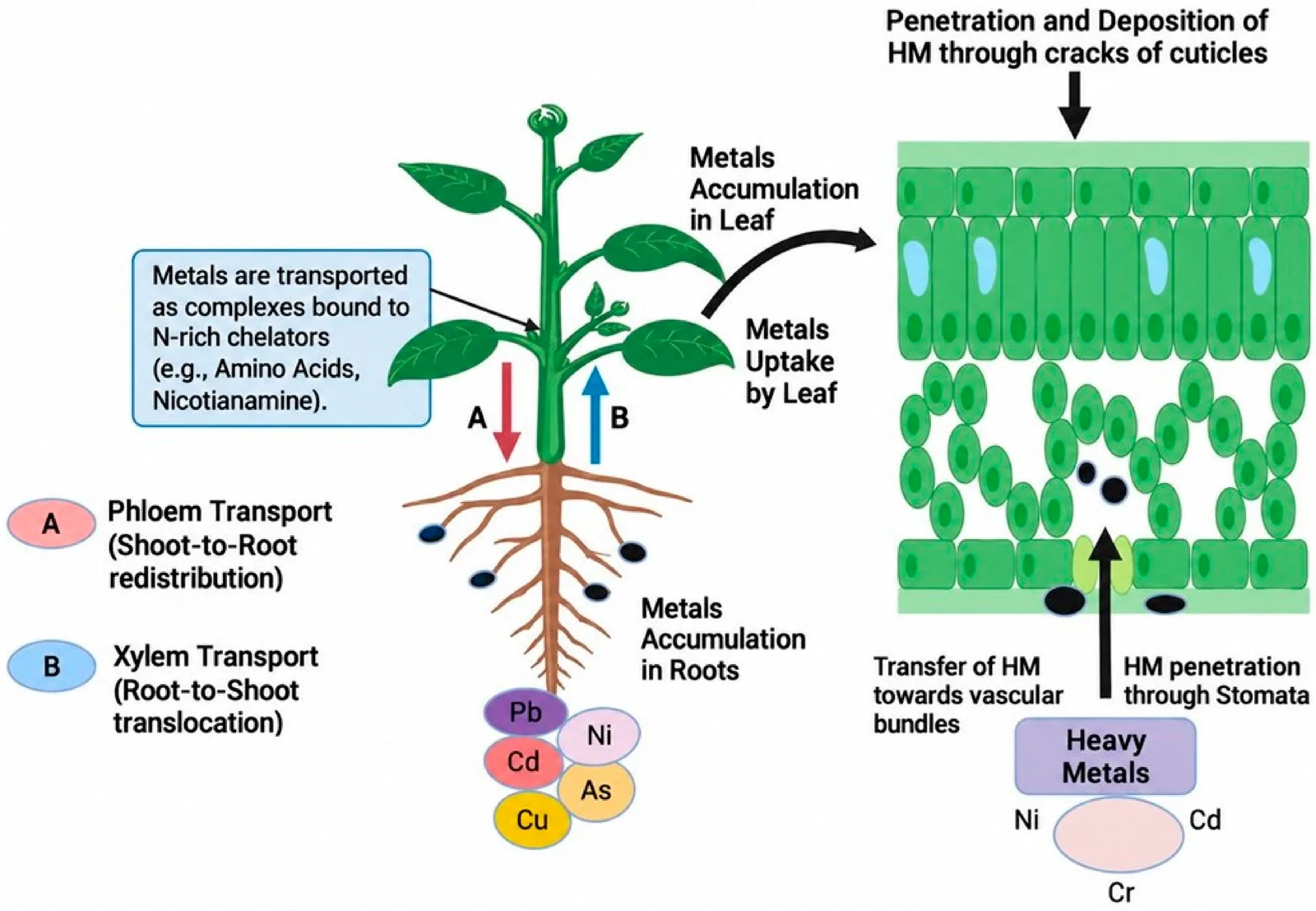
Anatomical pathways of heavy metal uptake and N-dependent vascular transport. Heavy metals enter the plant via root absorption from the soil or foliar uptake through the cuticle and stomata. Once internalized, systemic distribution occurs via bidirectional vascular transport. Root-to-shoot translocation (Arrow B) occurs in the xylem, while shoot-to-root redistribution (Arrow A) occurs in the phloem. This vascular movement requires heavy metals to be complexed with N-rich chelators (e.g., amino acids) to prevent oxidative damage to the transport tissues.

Once heavy metals enter the root, the plant must manage their systemic distribution. Plants transport heavy metals from the roots to the shoots (translocation) primarily through the xylem, and redistribute them from older leaves to developing tissues or roots through the phloem (Figure 5, Arrows A and B). Because free heavy metal cations are highly toxic to vascular tissues, this bidirectional movement relies heavily on N metabolism. Plants synthesize N-rich chelators, such as specific amino acids (e.g., histidine) and nicotianamine, which bind to the heavy metals to form stable, non-toxic complexes in the vascular sap [16]. Therefore, adequate N availability is required not only for cellular detoxification but also for the safe, systemic transport of metals, which is a critical mechanism for hyperaccumulator plants used in phytoextraction.

### 6.2. Management of Soil pH, Nutrient and Fertilizers

The management of soil pH and nutrient content is essential for the mitigation of heavy metals–N interaction in plants. Most heavy metals, such as Pb and Cd, can bind to plant roots, inhibit nutrient uptake, and cause toxicity [80,81,82,83]. In addition, some metals, such as Cu and Zn, can interact with N in soil, releasing toxic compounds. Proper soil management is required to prevent these interactions and reduce their negative impacts [84].

*Role of soil pH:* The availability of heavy metals and N in soil depends on a complex interaction of pH, redox conditions, organic matter, and soil mineralogy [85]. For cationic heavy metals, such as Cd^2+^, Pb^2+^, and Zn^2+^, solubility increases at acidic pH because hydrogen ions compete for binding sites on soil particles. Therefore, applying lime to raise the pH to neutral or slightly alkaline levels precipitates these cations as insoluble hydroxides or carbonates, reducing their bioavailability. However, this relationship is element-specific. For example, the metalloid arsenic (As) exists as an oxyanion. Arsenic mobility often increases at higher pH levels due to desorption from iron and aluminum oxides [16]. Furthermore, pH does not act alone. Soil organic matter provides chelating functional groups that bind metals, while clay mineralogy dictates the overall cation exchange capacity of the soil. Redox conditions also alter metal speciation; for instance, flooding soils lowers the redox potential and reduces sulfate to sulfide, which precipitates cadmium as highly insoluble cadmium sulfide (CdS) regardless of the baseline pH [85].

*Role of soil nutrients and fertilizers:* Soil nutrient content can also influence the interaction of HMs and N in plants. To reduce the availability of HMs, it is important to maintain adequate levels of soil nutrients, such as phosphorus (P) and potassium (K) [86]. P can help reduce the availability of Pb, Zn, and Cu in soil, while K can help reduce the availability of Ni and Cr [24,87].

The effect of N fertilizers on heavy metals availability depends on the chemical form of the applied N. The assumption that N fertilizers universally diminish heavy metals availability is incorrect. When plants absorb ammonium (NH_4_^+^), they excrete protons (H^+^) to maintain charge balance. This excretion acidifies the rhizosphere, which dissolves soil particles and increases the bioavailability of cationic heavy metals like Cd^2+^ and Zn^2+^ [88]. Conversely, when plants absorb nitrate (NO_3_^−^), they excrete hydroxyl ions (OH^−^) or bicarbonate. This excretion raises the rhizosphere pH, which promotes the precipitation of these metal cations and reduces their availability. Furthermore, the application rate of N fertilizers alters metal accumulation. High rates of N fertilization increase plant biomass and transpiration rates. This increased water movement can inadvertently increase the total mass flow of dissolved heavy metals from the soil into the root system [89]. Therefore, managing heavy metal toxicity requires selecting the appropriate N form (nitrate vs. ammonium) based on the specific metal contaminant and baseline soil pH, rather than simply applying bulk N.

### 6.3. Use of Organic Amendments

The use of organic amendments to mitigate heavy metal–N interaction in plants is a growing area of research that has been gaining attention in recent years. Organic amendments are an attractive option for managing heavy metal–N interactions in plants because they can reduce the availability of heavy metals and N to plants while providing beneficial nutrients [90]. These materials include compost, manure, straw, biochar, and other organic materials [91,92]. Biological strategies beyond the molecular level to mitigate the heavy metal–N interaction in diverse crops are shown in Table 3. Organic amendments can reduce the availability of heavy metals to plants by forming complexes with metal ions and binding them to soil particles. This reduces their ability to be taken up by plant roots [93]. Organic amendments can also increase the pH of soils, which reduces the availability of heavy metals [84,94].

*Reduction in availability of nitrogen:* Organic amendments alter the availability of N to plants, presenting a complex agronomic trade-off. Materials with a high carbon-to-N (C:N) ratio, such as straw or uncomposted biochar, stimulate soil microbes. These microbes consume available inorganic N, immobilizing it within the microbial biomass and making it temporarily unavailable for plant utilization [95]. While this immobilization prevents N leaching in over-fertilized soils, it is not intrinsically beneficial in contaminated environments. Heavy metal toxicity already damages root architecture and inhibits nitrate transporters, compromising the plant’s baseline ability to absorb N. Consequently, applying high C:N amendments can aggravate N limitation and increase overall plant stress. To manage this risk, agronomists often co-apply inorganic N fertilizers with organic amendments, or use low C:N materials like mature compost. This strategy ensures the plant receives adequate N for growth while the organic matrix successfully immobilizes the heavy metals.

Furthermore, the positive impact of organic amendments extends to improving soil health. These amendments can increase the soil microbial population. The rise in microbial activity enhances soil structure and promotes increased nutrient availability to plants. Additionally, it plays a crucial role in mitigating soil erosion, reinforcing the multifaceted advantages of incorporating organic amendments into soil management practices [96,97].

### 6.4. Multi-Omics and Genetic Engineering for Enhanced Phytoremediation

Recent advances in molecular tools provide new methods to understand and improve phytoremediation. Multi-omics technologies, integrating transcriptomics, proteomics, and metabolomics, reveal the complex regulatory networks linking heavy metal stress and N metabolism. Transcriptomic and metabolomic analyses show that plants exposed to heavy metals alter the expression of N transporters and amino acid synthesis pathways to produce metal-chelating compounds. For example, recent multi-omics profiling demonstrates that inoculating soils with specific plant-growth-promoting rhizobacteria (PGPR) alters the host plant’s transcriptome, upregulating N cycle genes to enhance cadmium phytoremediation [98]. This indicates that phytoremediation efficiency depends on the dynamic reprogramming of N metabolism at the whole-genome level.

In the soil environment, nanoparticle-based amendments increase phytoremediation efficiency [99]. Applying specific nanoparticles (e.g., zinc oxide or biochar-nanocomposites) to contaminated soils reduces heavy metal toxicity and restores N assimilation. Nanoparticles modulate ROS and improve the acquisition of essential nutrients like N under stress conditions [100]. Furthermore, these nanoparticles interact with the rhizosphere microbiome, promoting N-fixing bacteria that support hyperaccumulator growth. This suggests that combining nanotechnology with biological remediation provides a targeted approach to manage heavy metal bioavailability.

At the genetic level, CRISPR-Cas9 genome editing allows the precise manipulation of plant traits to improve heavy metal extraction and stabilization. Genetic engineering targets metal-binding proteins, such as metallothioneins, and metal transporters, such as the Heavy Metal ATPase (HMA) family. Recent advances use CRISPR-Cas9 to edit specific promoters, enhancing the root-specific expression of metal transporters without stunting overall plant growth [101,102,103]. This precision editing increases the accumulation of heavy metals in harvestable tissues while maintaining the N-use efficiency required for high biomass production. However, the field-scale application of these genetically engineered phytoremediators remains limited by ecological biosafety regulations regarding transgenic plants in open environments.

## 7. Conclusions

Heavy metals disrupt plant N assimilation by inhibiting key enzymes, such as nitrate reductase, and altering soil N cycling. Mitigating this toxicity requires managing both the external soil chemistry and the internal plant genetic response. While current research identifies several effective mitigation strategies, transitioning these approaches from controlled laboratory settings to agricultural fields presents specific challenges. For example, agronomic interventions like applying biochar or organic amendments successfully reduce heavy metal bioavailability by raising soil pH and chelating metal cations. However, materials with a high carbon-to-N ratio stimulate microbial activity, which temporarily immobilizes available soil N. In heavily contaminated soils where plant root systems are already damaged, this immobilization exacerbates N starvation. Similarly, phytoremediation faces biological constraints. While hyperaccumulator plants extract heavy metals efficiently, they typically produce low biomass and grow slowly. This makes phytoextraction a multi-year process, which is often economically unfeasible for commercial agriculture.

A significant research gap exists between laboratory models and field realities. Most studies evaluating heavy metal and N interactions use hydroponic systems or sterilized soils with single-metal exposures. These controlled conditions do not reflect actual agricultural environments, where soils contain multiple interacting heavy metals (e.g., Cd, Pb, and As simultaneously) and fluctuating moisture and N levels. Consequently, the synergistic or antagonistic effects of multi-metal contamination on N transport proteins remain poorly understood. Furthermore, while nanoparticle-based amendments show promise in greenhouse trials, their long-term ecological impacts on the soil microbiome and free-living diazotrophs remain unclear.

To address these gaps, future research must prioritize long-term field trials to evaluate how mitigation strategies perform under variable environmental conditions. To manage the complexity of field soils, researchers should integrate machine learning models to predict how specific soil amendments alter heavy metal bioavailability and N cycling across different soil types. Additionally, combining microbiome engineering (e.g., metal-tolerant, N-fixing bacteria) with genome-edited crops offers a pathway to improve phytoremediation efficiency. However, the deployment of these advanced biological tools requires clear regulatory frameworks to assess the ecological biosafety of introducing engineered organisms into open environments.

## Figures and Tables

**Table 1:** Mechanisms of heavy metal toxicity and plant responses diverse crops.

Crop	Heavy Metals	Plant Response	References
*Oryza sativa*	Cd, As	Inhibits photosynthesis; sensitive, reduced growth.	[35]
*Triticum aestivum*	Cd, Pb	Disrupts enzymes; tolerant, shoot accumulation.	[36]
*Glycine max*	Cd, Pb, Hg	Inhibits N-fixation; tolerant, root accumulation.	[37]
*Solanum lycopersicum*	Cd, Pb, Ni	Disrupts cell cycle; sensitive, stunted growth.	[38]
*Hordeum vulgare*	Cd, Pb, Cu	Impairs nutrient uptake; sensitive, reduced yield.	[39]
*Ipomoea aquatica*	Cd, Hg, Zn	Induces oxidative stress; high leaf accumulation.	[40]
*Brassica juncea*	Pb, Cd, Zn	Induces oxidative stress; hyperaccumulator (shoot).	[41]
Legumes/Cereals	Mo	Essential nutrient; enables N-assimilation.	[6]

**Table 2:** Key genes involved in N-mediated heavy metal transport and detoxification.

Gene ID	Plant Species	Target Heavy Metal	Specific Cellular Function
*OsFPN1*	*Oryza sativa*	Fe, Co, Ni	Mediates the efflux of metals from the cytosol to the vascular tissue to maintain homeostasis
*TaMTP8*	*Triticum aestivum*	Mn	Facilitates the vacuolar sequestration of excess manganese to prevent cytosolic toxicity
*AtPCS1*	*Arabidopsis thaliana*	Cd, Pb, As	Synthesizes phytochelatins (using N-rich glutathione) to chelate free heavy metal cations
*OsHMA3*	*Oryza sativa*	Cd, Zn, Pb	Transports metal-phytochelatin complexes across the tonoplast into the vacuole
*AtYSL1*	*Arabidopsis thaliana*	Cu, Fe, Zn	Transports metal-nicotianamine complexes across cell membranes for systemic distribution

**Table 3:** Non-molecular agronomic strategies for mitigating heavy metal toxicity and improving N utilization across different crops.

Crop	Mitigation Strategies	References
*Triticum aestivum*	Organic fertilizers, soil amendments, crop rotation	[70]
*Oryza sativa*	Phytoremediation, chelating agents, hydroponics	[71]
*Zea mays*	Organic fertilizers, soil amendments, crop rotation	[72]
*Glycine max*	Phytoremediation, chelating agents, hydroponics	[37]
*Arachis hypogaea*	Phytoremediation, chelating agents, hydroponics	[73]
*Solanum lycopersicum*	Organic fertilizers, soil amendments, crop rotation	[74]
*Solanum tuberosum*	Phytoremediation, chelating agents, hydroponics	[75]
*Daucus carota*	Organic fertilizers, soil amendments, crop rotation	[76]
*Spinacia oleracea*	Phytoremediation, chelating agents, hydroponics	[77]
*Brassica oleracea*	Organic fertilizers, soil amendments, crop rotation	[78]

## Data Availability

Data will be provided upon formal request.
